# Treatment of a Four-Rooted Maxillary Second Molar Detected with Cone-Beam Computed Tomography

**Published:** 2017-03

**Authors:** Nahid Mohammadzade Akhlaghi, Mahta Fazlyab

**Affiliations:** 1 Associate Professor, Department of Endodontics, Dental Branch, Islamic Azad University, Tehran, Iran; 2 Assistant Professor, Department of Endodontics, Dental Branch, Islamic Azad University, Tehran, Iran; 3 Iranian Center for Endodontic Research, Research Institute of Dental Sciences, Shahid Beheshti University of Medical Sciences, Tehran, Iran

**Keywords:** Cone-Beam Computed Tomography, Root Canal Therapy, Tooth Root

## Abstract

The significance of clinician’s knowledge about root canal anatomy and its possible variations cannot be overlooked. In some cases, taking advantage of complementary imaging techniques can help achieve a perfect flawless endodontic treatment. This article reports endodontic management of a second maxillary molar that had an uncommon anatomy of the chamber floor. After obtaining a cone-beam computed tomography (CBCT) image, the presence of a second palatal root was confirmed. All four roots were treated and patient’s symptoms were resolved.

## INTRODUCTION

Successful root canal therapy requires a thorough knowledge of root and root canal morphology. The hard tissue armor of the dental pulp takes numerous configurations. A thorough knowledge of tooth morphology, careful interpretation of radiographic images, proper access preparation and a detailed exploration of the inside of the tooth are essential prerequisites for a successful treatment outcome [1].

Endodontic management of maxillary second molars (MSMs) can sometimes be very challenging due to complex anatomy of the roots. The majority (88.6%) of MSMs have three distally inclined roots [2] and compared to the maxillary first molars the roots tend to be closer [3] and fusion of all or two roots is more likely (25.8%) [2,4]. The mesiobuccal (MB) root has equal incidence of one or two canals. The distobuccal (DB) root usually contains one canal and the palatal (P) canal is broad in mesiodistal direction and more frequently contains a single canal, as well [4]. According to Sert and Bayirli [5], the frequency of single canals in DB and P roots is over 99%. However, there are many reported variations in root morphology that include four-rooted MSMs with either two P roots containing single canals [6–8], two DB single-canal roots [9–11] or two separate MB roots [12], presence of 5 roots and root canals (double P and double DB roots) [13] or an uncommon morphology resembling mandibular molars without P canal and two mesial and distal roots containing two canals each [14,15]. The presence of a single root and a single canal has also been reported [2]. According to Christie et al*.* [16] MSMs have the highest occurrence of two P canals in double P roots. This anatomy mostly included two forms; two long and divergent P roots and two short parallel P roots resembling their buccal counterparts [16]. The fourth root is called “radix paramolaris” which is defined as a supernumerary structure in third or second molars [17].

Since the introduction of cone-beam computed tomography (CBCT), when 3D imaging is necessary, this technology is considered the standard of care by some authors [18]. Although it is originated from conventional medical computed tomography (CT), CBCT differs in a number of fundamental ways which improve its suitability for dental imaging [19]. In maxillary molars especially in MSMs, superimposition of anatomical structures (zygomatic arch) over the roots is very likely [20]. Conventional angled radiographs, at their best, can only partially reveal these configurations. Clinicians have benefited from 3D imaging by means of CBCT in terms of detection of uncommon anatomy of MSMs during endodontic management [10,15,21].

The purpose of this article was to investigate different anatomical variations and more specifically the number of roots in MSMs with special emphasis on the occurrence of four roots. A case of endodontic therapy in a four-rooted MSM including two P roots is also described. Navigation of all roots and canals facilitated by 3D CBCT imaging led to successful treatment of this tooth.

## CASE REPORT

**Fig. 1:**
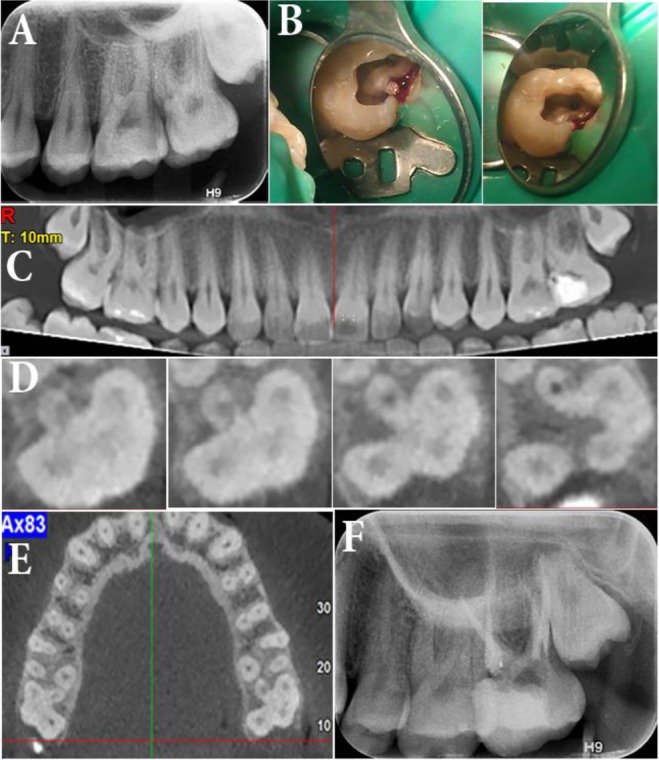
(A) Pretreatment image of the left second maxillary molar with deep cavity. Note the unusual shape of the roots. (B) Preparation of the access cavity and irregular form of the pulp chamber. (C) and (D) Cone-beam computed tomography image of the tooth which reveals the presence of four separate roots. (E) Note the second molar in the opposite quadrant which also has four roots. (F) Post-endodontic image that shows treatment of all four roots.

An 18 year-old female with no systemic condition presented to a private dental clinic complaining of severe hyper-sensitivity of her maxillary left second molar. On clinical inspection, the tooth had a class I composite filling without marginal discoloration. A lucency of enamel was obvious beneath the mesial marginal ridge. A diagnostic parallel image showed a mesial carious lesion in the second molar and with careful inspection the palatal root was not well-defined which was justifiable due to the superimposition of the zygomatic arch (Fig. 1A). According to the patient, she had been tolerating extreme tooth hyper-sensitivity lately and it led to severe nocturnal pain during the last two nights; upon cold testing with Endo-Ice frozen gas (Coltene/Whaledent, Inc., Mahwah, NJ, USA) severe discomfort was reported by the patient. The treatment plan (root canal therapy) was explained to the patient and an informed consent was obtained from her.

After local anesthesia with buccal infiltration of 2% lidocaine with 1:80,000 epinephrine (Darupakhsh, Tehran, Iran) and tooth isolation with rubber dam, access cavity was prepared and caries was removed. Three canals were detected; upon inspection, the chamber floor had an uncommon anatomy as the MB and DB canals were abnormally far away from each other with almost 3mm distance. Also, the P canal was located too mesially so that it could be called the mesiopalatal (MP) canal; with more careful inspection, the orifice of another canal in the distopalatal (DP) area was detected (Fig. 1B).

After working length determination with an electronic apex locator (Root ZX; J. Morita USA, Inc., Irvine, CA, USA), cleaning and shaping of the four canals were accomplished with ProTaper rotary instruments (Dentsply Maillefer, Ballaigues, Switzerland) up to F2 (25/0.08). Then, the canals were filled with calcium hydroxide paste (Golchay, Tehran, Iran) mixed with saline. The tooth was temporarily restored. For more accurate evaluation of the tooth anatomy, the patient was referred for CBCT.

On panoramic view (Fig. 1C) nothing noteworthy was detected regarding the root anatomy of the left second molar. However, in axial planes from the chamber floor to the more apical levels, the presence of two P canals (DP and MP) was confirmed (Fig. 1D).

**Fig. 2:**
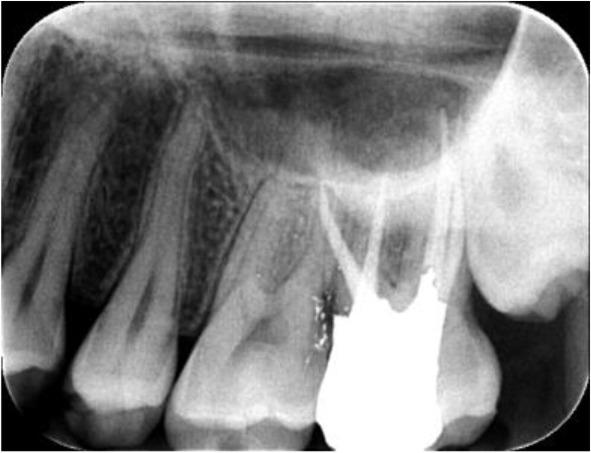
Canal filling following RCT

**Fig. 3:**
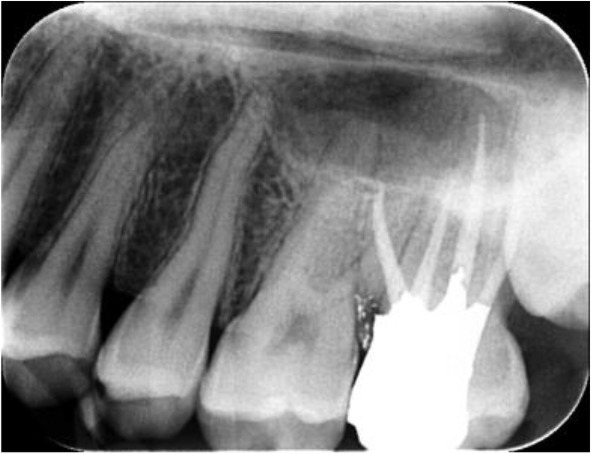
After six months of follow-up

In the axial plane of the whole jaw, the right MSM also had a similar anatomy to that of its left counterpart (Fig. 1E) and the patient was informed of this fact. On the second appointment, the root canal treatment of the tooth was completed and the patient was referred for tooth restoration (Fig. 2). The six-month follow-up session showed that the tooth was completely asymptomatic and in function (Fig. 3).

## DISCUSSION

This report presented successful treatment of a MSM with two separate P roots that were parallel and had comparable length to their buccal counterparts. The CBCT images facilitated the diagnosis of the root anatomy.

It is generally accepted that in the most frequent form, MSMs have three roots with lower incidence of second MB canal than maxillary first molars [5]. An anomalous and infrequent root morphology, such as double P roots in the maxillary molars, is rarely mentioned in textbooks [16]. In 1991, the least frequent anomaly of MSM appeared to be double P roots [16]. However, evaluation of radiographs by Pineda and Kuttler [22] in 1972 revealed the prevalence of four roots in MSMs to be 35% and Vertucci [23] in 1984 reported that 8–12% of extracted decalcified MSMs had four root canals that were always located in the MB root. Libfeld and Rotstein [24] in 1989 evaluated the radiographs of 1000 MSMs and stated that 0.4% of teeth had four distinct roots. The prevalence of three, two and single roots was reported to be 90.6%, 6% and 3%, respectively. These variable results can be due to the different assessment methods (from in vitro direct evaluation to radiographic assessment).

It is noteworthy that in 1970s the methods for assessment of tooth morphology and internal anatomy included direct observation of the extracted tooth with/without armed eye, macroscopic/microscopic sections, transverse sections and micrometric measurements, filling and decalcification, filling and clearing and grinding and radiography that all were limited to in vitro examination models [22]. The only observational method that could be clinically used was taking radiographs [2,16]. However, another problem was that the image of maxillary molars is often superimposed with the zygomatic bone and the zygomatic process of the maxilla over the roots, especially when the radiograph is taken with the bisecting angle technique [20]. Overall, in order to detect an uncommon anatomy such as four roots the teeth had to be either extracted and evaluated or the chance of radiographic detection was not very high. Since the introduction of CBCT to dentistry, endodontists have benefited from the 3D images that enable evaluation of teeth from different aspects [18]. The number of case reports about the detection of extra roots/canals in these teeth has raised since then [13,15,25].

The last but not least is the possibility of ethnical tendency for different anatomical variations. As the only national survey in this regard, Rouhani et al. [26] conducted a CBCT assessment on 250 extracted maxillary molars with equal numbers of first and second molars.

The canal configuration was evaluated in three P, DB and MB roots and the presence of extra roots such as second P or MB canal was reported to be 0.8–16% and limited to MSMs. There are three reports of MSMs with double P roots [25,27,28] and one report of three separate B roots [12] in the Iranian patients from Shiraz [25], Tehran [28], Mashhad [12,27] and Tabriz [29]. It can be concluded that due to distribution of these reports in almost all areas of Iran, and considering the ∼18% prevalence of reported cases of MSMs with extra roots being from Iran, there is space for a national investigation with large sample size to find the frequency of extra roots in MSMs among the Iranian population.
